# Postbiotics of Marine Origin and Their Therapeutic Application

**DOI:** 10.3390/md23090335

**Published:** 2025-08-24

**Authors:** Isabel M. Cerezo, Olivia Pérez-Gómez, Sonia Rohra-Benítez, Marta Domínguez-Maqueda, Jorge García-Márquez, Salvador Arijo

**Affiliations:** 1 Departamento de Microbiología, Facultad de Ciencias, Instituto Andaluz de Biotecnología y Desarrollo Azul (IBYDA), CEIMAR, Universidad de Málaga, Campus Universitario de Teatinos s/n, 29071 Málaga, Spain; cerezoi@uma.es (I.M.C.); olipergom@uma.es (O.P.-G.); soniarb@uma.es (S.R.-B.); martadm@uma.es (M.D.-M.); j.garcia@uma.es (J.G.-M.); 2 Departamento de Biología y Geología, Escuela Superior de Ingeniería, CEIMAR, Universidad de Almería, 04120 La Cañada de San Urbano, Spain

**Keywords:** antimicrobial activity, bacteria, extracellular products, immunomodulation, microbiota, probiotic

## Abstract

The increase in antibiotic-resistant pathogens has prompted the search for alternative therapies. One such alternative is the use of probiotic microorganisms. However, growing interest is now turning toward postbiotics—non-viable microbial cells and/or their components or metabolites—that can confer health benefits without the risks associated with administering live microbes. Marine ecosystems, characterized by extreme and diverse environmental conditions, are a promising yet underexplored source of microorganisms capable of producing unique postbiotic compounds. These include bioactive peptides, polysaccharides, lipoteichoic acids, and short-chain fatty acids produced by marine bacteria. Such compounds often exhibit enhanced stability and potent biological activity, offering therapeutic potential across a wide range of applications. This review explores the current knowledge on postbiotics of marine origin, highlighting their antimicrobial, anti-inflammatory, immunomodulatory, and anticancer properties. We also examine recent in vitro and in vivo studies that demonstrate their efficacy in human and animal health. Some marine bacteria that have been studied for use as postbiotics belong to the genera *Bacillus*, *Halobacillus*, *Halomonas*, *Mameliella*, *Shewanella*, *Streptomyces*, *Pseudoalteromonas*, *Ruegeria*, *Vibrio*, and *Weissella*. In conclusion, although the use of the marine environment as a source of postbiotics is currently limited compared to other environments, studies conducted to date demonstrate its potential.

## 1. Introduction

Infectious diseases remain one of the most significant challenges to global health, affecting not only humans but also a wide range of domestic and wild animals. They are caused by diverse pathogenic agents, including bacteria, viruses, fungi, and parasites, and can lead to acute outbreaks or chronic conditions with serious economic, social, and environmental repercussions [1,2].

In humans, diseases such as tuberculosis, influenza, malaria, and dengue continue to contribute to substantial morbidity and mortality, particularly in low- and middle-income countries [3]. The global burden of zoonotic infections—diseases transmitted between animals and humans—adds further complexity [4]. Zoonotic pathogens are estimated to account for more than 60% of emerging infectious diseases [5]. In veterinary contexts, infectious diseases in livestock, aquaculture, and companion animals compromise animal health, welfare, and productivity [6]. Outbreaks of bacterial infections (e.g., colibacillosis in poultry, mastitis in dairy cattle), viral diseases (e.g., African swine fever, avian influenza), and parasitic infestations result in significant economic losses and threaten food security [7,8]. In aquaculture, intensive farming conditions have facilitated the spread of pathogens such as *Vibrio*, *Photobacterium*, and *Aeromonas*, and viral agents like nodaviruses and iridoviruses [9,10]. Several drivers exacerbate the emergence and re-emergence of infectious diseases, including intensification of farming systems and high stocking densities; global trade of live animals and animal-derived products; environmental changes and ecosystem disruption; and antimicrobial misuse and the emergence of resistant strains [11,12,13,14].

To control infectious diseases, antibiotic treatments have historically been implemented. However, widespread antibiotic use and failures in infection prevention and control strategies have contributed to the emergence and spread of antibiotic resistance, posing a significant global health threat [15,16]. Antimicrobial resistance (AMR) is now considered one of the most serious threats to public health worldwide [17]. The World Health Organization (WHO) has classified AMR among the top ten global public health threats [16].

In this sense, in 2024, the World Health Organization (WHO) published the list of global priority pathogens—a catalogue of 15 groups of bacteria categorized into three priority tiers based on their antibiotic resistance levels: critical (*Acinetobacter baumannii*, carbapenem-resistant; Enterobacterales, third-generation cephalosporin-resistant; Enterobacterales, carbapenem-resistant; *Mycobacterium tuberculosis*, rifampicin-resistant), high (*Salmonella typhi*, fluoroquinolone-resistant; *Shigella* spp., fluoroquinolone-resistant; *Enterococcus faecium*, vancomycin-resistant; *Pseudomonas aeruginosa*, carbapenem-resistant; non-typhoidal *Salmonella*, fluoroquinolone-resistant; *Neisseria gonorrhoeae*, third-generation cephalosporin and/or fluoroquinolone-resistant; *Staphylococcus aureus*, methicillin-resistant), and medium (Group A Streptococci, macrolide-resistant; *Streptococcus pneumoniae*, macrolide-resistant; *Haemophilus influenzae*, ampicillin-resistant; Group B Streptococci, penicillin-resistant) [18]. These pathogens present serious challenges due to their resistance to conventional antibiotics.

The problem of AMR is equally important and prevalent in animals, although it is emphasized to a lesser extent [19]. Antimicrobial agents are being employed for food animal production either as therapeutic, metaphylactic, or prophylactic agents or as growth promoters [20]. As an outcome of extensive public health concerns regarding antimicrobial growth promoter (AGP) usage in livestock, the European Union progressively banned all AGPs in the livestock industry [21]. The emergence and spread of drug-resistant bacteria arise from a myriad of ecological and evolutionary interacting factors, either natural or human-driven.

The One Health approach has been adopted internationally as a framework to address this challenge [22]. One Health recognizes that human health, animal health, and ecosystem health are interconnected and require coordinated actions across disciplines and sectors. This approach promotes (i) judicious use of antibiotics in all settings; (ii) enhanced biosecurity and hygiene practices; (iii) surveillance systems that integrate human, animal, and environmental data; and (iv) investment in the development of effective alternatives to antibiotics. Without urgent action, it is estimated that by 2050, antimicrobial resistance could cause up to 10 million deaths and cumulative economic damage exceeding USD 100 trillion [23]. Thus, the rise of AMR has led to intense interest in non-antibiotic interventions. Each strategy has strengths and limitations, and most will likely be used in combination under a One Health framework [24,25]. In this context, natural products play a crucial role in the development of new drugs [26]. Among the diverse sources being investigated for preventive and therapeutic measures, marine ecosystems have emerged as a key focus [27].

## 2. The Use of Probiotics and Their Limitations

Probiotics have been highlighted as a promising strategy to mitigate the challenges associated with antibiotic overuse by offering several health benefits. Probiotics are defined as “live microorganisms that, when administered in adequate amounts, confer a health benefit on the host” [28]. To qualify as a probiotic, a strain must exert positive effects on host health, remain viable throughout its shelf life, and pose no safety risks [29].

Despite these advantages, the administration of live microbial cells carries potential risks [30]. In immunocompromised hosts, for example, probiotics can exacerbate inflammation by overstimulating immune responses [31]. Furthermore, ensuring consistent microbial survival throughout the shelf life of probiotic products can be challenging, and viability may decline during storage or processing [32]. Live probiotics may also engage in horizontal gene transfer, potentially spreading antibiotic-resistance or virulence genes to other microorganisms [33]. Opportunistic infections represent another concern: adhesive probiotic strains that persist on the intestinal mucosa can translocate across a compromised epithelial barrier, leading to bacteremia, especially under conditions of increased gut permeability or invasive procedures [30,34,35].

Regulatory frameworks for probiotics remain uneven and sometimes ambiguous. Although there is broad agreement on fundamental safety principles, there is little consensus on the specific studies or methodologies required to validate probiotic safety and efficacy [36]. Consequently, different countries maintain divergent legal requirements for strain characterization, manufacturing standards, and permitted health claims [37,38,39]. This regulatory heterogeneity complicates international trade and hampers the global adoption of probiotic interventions in human and livestock production systems.

Given these challenges—viability concerns, safety risks, and regulatory variability—research has increasingly turned toward alternatives derived from probiotic cells that retain health-promoting functions.

## 3. Use of Postbiotics for Health Management

Given the diversity of terms historically used to describe non-viable microorganisms or their derivatives with health-promoting properties, the International Scientific Association for Probiotics and Prebiotics (ISAPP) proposed a standardized definition to unify the terminology. In 2021, postbiotics were defined as “preparations of inanimate microorganisms and/or their components that confer a health benefit on the host” [29]. This definition encompasses both whole inactivated microbial cells and their associated metabolites or structural components. Despite this consensus, both “postbiotic” and “paraprobiotic” remain in use across the scientific literature, reflecting ongoing debate and transitional adoption.

Postbiotic preparations can include a wide array of bacterial derivatives, such as cell wall fragments, membrane lipids, exopolysaccharides (EPSs), short-chain fatty acids (SCFAs), peptides, proteins, and soluble bioactive metabolites—all of which are known to exhibit diverse biological activities [40,41,42]. The production of postbiotics involves various methods depending on the nature of the compound targeted. Common techniques include centrifugation and filtration (typically through 0.45 µm and 0.22 µm membranes) [43,44]. Another common approach is cell lysis, which can be induced through physical techniques like sonication and homogenization, as well as freeze–thaw cycles, while chemical methods include detergents and enzymes [45,46]. Less frequently employed techniques involve the use of organic solvents, such as methanol or ethanol, for the extraction of lipids or other hydrophobic molecules. Moreover, the postbiotic sample concentration for advanced analysis can be achieved through lyophilization, also known as freeze drying [39].

Despite the limited understanding of the complete biochemical composition of many postbiotic formulations, recent advances in proteomics and metabolomics have identified numerous bioactive molecules [47,48,49]. These include antimicrobial peptides, cyclic lipopeptides (e.g., surfactin, fengycin, pumilacidin), SCFAs, and various polysaccharides. For example, surfactin variants from *Bacillus* strains have shown strong antibacterial and antifungal activities against a wide range of pathogens, including *Vibrio alginolyticus*, *Staphylococcus aureus*, *Escherichia coli*, and *Magnaporthe grisea* [50,51,52,53]. The focus of the research is currently on the analysis of isolated compounds [44].

Postbiotics offer several advantages over probiotics: (i) reduced risk of translocation and infection, (ii) improved shelf stability, and (iii) better predictability in industrial applications [54]. However, it must be acknowledged that their effects are sometimes weaker than those of live probiotics, depending on the strain and [55].

**Figure 1:**
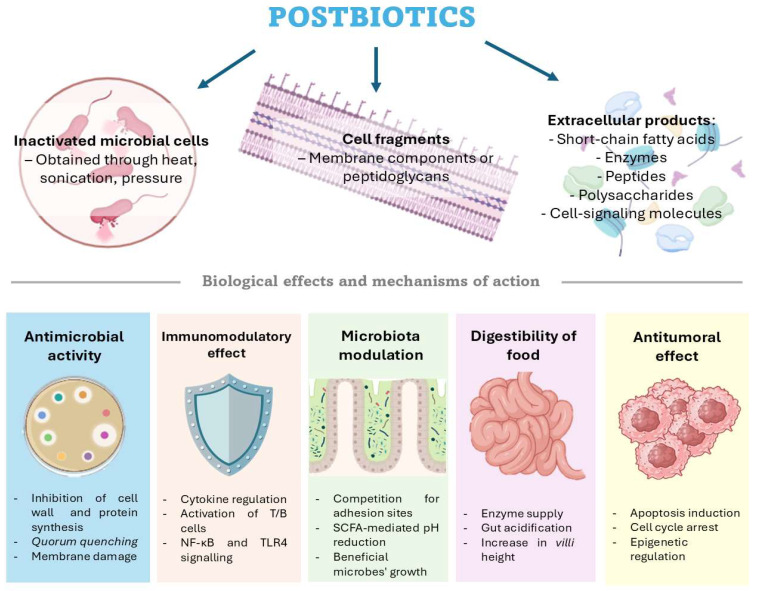
Different types of postbiotics and their primary biological effects and mechanisms of action. Figure created by the authors using https://BioRender.com.

The scope of postbiotic applications is broad (Figure 1), encompassing antimicrobial, anti-inflammatory, antioxidant, and immunostimulatory activities, as well as microbiota modulation and epithelial barrier reinforcement [56]. These functionalities can currently be employed in different areas, such as in medicine and food [57] and the livestock or aquaculture industry [58]. Its application in medicine covers different areas; for example, the antitumoral effect against different types of cancers, such as colorectal, lung, and liver cancers, among others, is being studied. In addition to cancer, it has other applications, such as modulation of microbiota with a reduction in harmful microorganisms, antioxidant properties, and anti-inflammatory effects, for example, in diseases like colitis; these studies have been carried out both in humans and animals [57].

### 3.1. Antimicrobial Activity

An important function of postbiotics is their ability to control pathogenic microorganisms by inhibiting their growth, interfering with biofilm development and swarming behavior, and limiting their capacity to colonize the host [59,60]. The effectiveness of these compounds is largely determined by their biochemical composition, which can vary depending on the culture conditions and the susceptibility of the host organism they interact with [61]. Some compounds operate by inhibiting cell wall production; however, others show varied modes of action to regulate microbial growth, including interfering with protein synthesis or communication systems like quorum sensing, blocking DNA replication, and breaking the cell membrane.

Bacteriocins are peptides produced by some bacteria to inhibit the growth of other competing bacteria. These bacterins have been used as postbiotics to control certain diseases, such as tuberculosis [62] and diseases produced by *Clostridioides difficile* [63], *Staphylococcus aureus* [64], *Listeria monocytogenes* [65], *E. coli*, *Salmonella enterica* [66], *Streptococcus pneumoniae*, and *Haemophilus influenzae* [67].

Among microbial products with notable antibacterial effects are SCFAs, mostly acetate, propionate, and butyrate [68,69]. They prevent pathogenic bacteria by damaging cell membranes and reducing the interior pH, thereby causing metabolic stress and cell death [70]. SCFAs also acidify the surroundings, restricting pathogen development and modifying the host immune response to strengthen barrier defenses [70]. Because of these characteristics, SCFAs show promise as functional components for food and agricultural uses and as therapeutic agents in infection management.

### 3.2. Immunomodulatory Effect

Postbiotics are involved in the immune response and include SCFAs, exopolysaccharides (EPSs), lipopolysaccharides, and even peptidoglycans. Among these immunomodulatory drugs, SCFAs, which include butyrate, propionate, and acetate, have shown advantageous effects in aquatic organisms like zebrafish (*Danio rerio*), European sea bass (*Dicentrarchus labrax*), yellow catfish (*Pelteobagrus fulvidraco*), gilthead seabream (*Sparus aurata*), largemouth bass (*Micropterus salmoides*), and ridgetail white prawn (*Exopalaemon carinicauda*) [71,72,73,74]. In these species, dietary supplementation with SCFAs has been connected with greater antioxidant enzyme activity, high levels of antioxidants, including SOD, CAT, and GSH-Px, and the upregulation of immune markers like TNF- and IL-8 [75].

In mammalian models, including humans and mice, SCFAs stimulate B cell and T-helper (Th) lymphocyte activity and, hence, not only support innate immune responses but also improve adaptive immunity [76,77]. Given their great immunoregulatory capabilities, SCFAs are regarded as promising candidates for preventive health measures. For example, butyrate increases retinoic acid production by inhibiting HDAC3 in epithelial cells from both human and mouse lines [78]. Similarly, propionic acid (PA) has been shown to offer neuroprotection and boost T cell numbers in the setting of neurodegeneration linked to multiple sclerosis [79].

Some postbiotics, which are Gram-negative bacteria, contain lipopolysaccharides (LPSs) with mild immunomodulatory properties. These LPSs are often employed as vaccine adjuvants or to activate innate immune responses [80,81]. While the general structure of LPSs—comprising lipid A, a core oligosaccharide, and an O-antigen—is conserved, the lipid A region exhibits significant variability among marine bacteria [82], such as *Marinomonas vaga* ATCC 27119, *Pseudoalteromonas*, *Cellulophaga pacifica*, and *Vibrio fischeri* [83,84,85,86]. These structural differences are closely linked to the capacity of Gram-negative bacteria to evade detection by the host’s innate immune system [86]. In humans, LPSs interact with the TLR4 receptor on macrophages, leading to the activation of transcription factors like NF-κB and IRFs [87,88]. Although TLR4 has been identified in various fish species, including *Labeo rohita*, *Danio rerio*, and *Ctenopharyngodon idella* [89,90,91], its signaling pathways in fish often diverge from those observed in mammals [92,93]. Moreover, TLR4 is not the predominant immune receptor in fish. Other pattern recognition receptors, such as TLR5, scavenger receptors (SRs), and the nucleotide-binding oligomerization domain (NOD), play more roles in immune sensing and response [94,95].

### 3.3. Microbiota Modulation

Symbiotic microorganisms associated with a host play a crucial role in the survival and overall health of animals [96]. The balance of this microbial ecosystem, which coexists in symbiosis with the host, is essential for maintaining physiological functions and immune homeostasis. In addition to their metabolic and immunological roles, certain biotic derivatives can significantly contribute to the integrity of the intestinal barrier by supporting microbial equilibrium [97]. Among the various factors influencing the gut microbiota, diet stands out as a key modulator.

The inclusion of postbiotics in the diet has been shown to directly affect microbial populations. For instance, enzymes and supernatant cells from *Lactiplantibacillus plantarum* have been found to promote the proliferation of beneficial microbes such as Firmicutes, while suppressing harmful ones like Proteobacteria in chickens [98]. Similarly, administration of heat-inactivated *Ligilactobacillus salivarius* strain 189 (HK LS 189) to pigs altered the microbial composition, decreasing *Prevotella* and increasing *Parabacteroides*, which was associated with reduced growth performance [99]. This effect may be due to the ability of postbiotics to modulate the microbiota by altering the intestinal environment. Compounds such as short-chain fatty acids, like butyrate, propionate, and acetate, lower the luminal pH, inhibiting the growth of pathogenic microorganisms while favoring the expansion of beneficial bacteria [100,101].

In addition, certain surface proteins, exopolysaccharides, and bacteriocins produced by probiotic strains such as *Lactiplantibacillus plantarum* NCU116 or *Lactobacillus gasseri* LA39 have been shown to bind competitively to the intestinal epithelium, thereby preventing pathogen adhesion and colonization [102]. These interactions contribute to the maintenance of microbial homeostasis.

### 3.4. Digestibility of Food

For humans and livestock alike, evidence is accumulating that postbiotic supplementation boosts the breakdown of dietary constituents and nutrient absorption. Mechanistically, postbiotics improve digestibility through multiple complementary pathways. First, many microbes produce *exo*-enzymes (amylases, proteases, lipases) during fermentation, and these can remain active in the postbiotic. For example, *Bacillus* species—widely used in poultry and pigs as probiotics—secrete a variety of digestive enzymes in the gut, which directly enhance the breakdown of starches and proteins [103,104]. Even *Bacillus* postbiotics can contain residual enzymes or enzyme-inducing factors that augment host enzyme activity [105]. Second, postbiotic acids (e.g., lactate, acetate) acidify the gut lumen, which can optimize the solubility and enzymatic activity of nutrients. In ruminants, mild pH reduction by microbial acids can favor fiber-fermenting bacteria and hasten carbohydrate digestion. Third, peptides and SCFAs in postbiotics serve as energy sources for enterocytes and stimulate the gut surface area [106]. Notably, postbiotics have been reported to increase the intestinal villus height and mucosal development in animals, which expands the absorptive surface [107].

In ruminants, postbiotic supplements have repeatedly improved nutrient utilization and growth. In dairy cows, providing a lactobacilli-derived postbiotic before and after calving raised the feed intake and ruminal fermentation, yielding greater total-tract digestibility of dry matter (DM), organic matter (OM), and neutral detergent fiber (NDF) [108]. Likewise, lambs consuming an *L. plantarum* postbiotic showed higher daily weight gain without increasing their feed intake, precisely because their DM, CP, and NDF digestibility were significantly higher than that of controls [109]. The lambs also had increased ruminal propionate levels, indicating more efficient carbohydrate conversion. In monogastric farm animals, although fewer direct measurements of “digestibility” exist, the evidence is consistent: postbiotic-fed pigs and chickens grow faster than those receiving a postbiotic-free diet, which implies better feed use [110,111]. Indeed, multiple broiler studies find that *Bacillus*- or lactobacilli-based postbiotics improve feed conversion ratios and body weight gain [112]. Overall, by supplying microbial enzymes, lowering the luminal pH, and enhancing the epithelium status, postbiotics create a more efficient digestive environment.

### 3.5. Antitumoral Effects

As previously discussed, postbiotics have demonstrated potential antitumor properties across various cancer types, acting at multiple molecular levels. Compounds associated with these effects include SCFAs, proteins, enzymes, and peptides, among others [113].

Notably, SCFAs such as butyrate have been shown to induce cell cycle arrest and apoptosis in several cancer cell lines, including those derived from colorectal, cervical, and breast cancers [113,114,115]. These effects have been observed with postbiotic compounds derived from strains belonging to the genera *Lactobacillus* and *Bifidobacterium* [113], among others. For example, in a study conducted by the authors Asoudeh-Fard et al. (2024) [116], extracellular products from *Lactobacillus fermentum* exhibited cytotoxic effects on the cervical cancer cell line, HeLa, by promoting apoptotic cell death.

## 4. The Marine Environment as a Source of Postbiotics

Oceans, which cover more than 70% of the planet’s surface, are characterized by their high biodiversity and ecological variability, making them a promising source for the discovery of novel bioactive compounds [117]. Marine environments encompass a wide range of physicochemical conditions, which have driven the evolution of unique adaptive strategies in marine organisms [118]. The marine environment ranges from nutrient-rich areas to regions with limited nutritional availability, where only certain specialized organisms can survive. The extreme conditions that characterize these ecosystems, such as variations in salinity, high pressure, changes in temperature, and light availability, contribute to marine microorganisms producing metabolites that differ from those found in terrestrial areas. These unique features have driven the search for ways to utilize derivatives from these marine microorganisms, with potential commercial applications. For example, reverse transcriptase isolated from *Hypericum perforatum* (Guttiferae) exhibits activity against the structure of retroviruses’ capsid, including HIV [119], and compounds, such those derived from the marine bacterium *Salinispora tropica*, are in phase clinical trials due to their anticancer, antimicrobial, and analgesic activity [120,121].

According to the Global Biodiversity Assessment conducted by the United Nations Environment Programme, marine species account for approximately 178,000 known organisms spanning 34 phyla [122]. Microorganism adaptive capacity, coupled with the rich taxonomic diversity of the marine biosphere, positions oceanic microorganisms as a valuable and largely underexplored source of biologically active metabolites. Such diversity offers the potential for discovering compounds with therapeutic or industrial relevance [123]. Although this field is still in an early stage of exploration, the vast genetic and biochemical diversity of marine microorganisms positions them as a promising source for yet unexplored applications. These are frequently used in aquaculture.

The utilization of postbiotics of marine origin remains an area of research that has yet to be extensively explored. A greater volume of research has been dedicated to the field of marine probiotics, with a particular focus on the individual components that comprise these organisms.

## 5. Postbiotics Isolated from the Aquatic Environment

Although most bacteria used as probiotics and postbiotics are Gram-positive, the marine environment is characterized by a greater abundance of Gram-negative bacteria [124,125]. Gram-positive bacteria are more frequently found in marine sediments [126], while the microbiota of fish is largely composed of Gram-negative taxa [127]. Despite this microbial richness, the exploration of marine microorganisms with postbiotic capacity remains limited, with the majority of research focusing on their application in aquaculture. This section provides a brief overview of some of the bacteria isolated from the aquatic environment and their use as postbiotics. A summary of their type of compound, extraction method, and activities can be found in Table 1.

Among Gram-positive marine bacteria, the genus *Bacillus* is one of the most extensively studied for its probiotic and postbiotic potential. *Bacillus amyloliquefaciens* COFCAU_P1 has demonstrated both antimicrobial and antioxidant properties through its postbiotic metabolites, effectively suppressing various harmful bacterial strains [128]. Additionally, these postbiotics enhanced immune responses in *Labeo rohita* by stimulating immune cell activity and promoting the expression of cytokines such as IL-1β, IL-10, IFN-γ, and TNF-α, aligning with findings from a probiotic in vivo assay [129]. Importantly, administration of these postbiotics has been shown to be safe, with no adverse effects reported. In a separate study, the sponge *Lamellodysidea herbacea* served as the source for the isolation of *B. amyloliquefaciens* MK135790, as well as *Alcaligenes faecalis* MK135791 [130]. These strains exhibited antimicrobial activity against a wide range of human pathogens. Ethyl acetate-extracted metabolites of the strain *B. amyloliquefaciens* MK135790 showed significant activity against four pathogens, *B. cereus*, *B. subtilis*, *S. aureus*, and *S. enterica typhimurium*, while Alcaligenes faecalis metabolites inhibited the growth of the pathogens *B. subtilis*, *E. coli*, and *S. enterica typhimurium* [129].

*Bacillus pumilus* H2, isolated from marine sediment, produces an anti-*Vibrio* compound obtained from cell-free supernatant and purified using reversed-phase HPLC [131]. This substance showed the ability to inhibit several *Vibrio* strains at a minimum inhibitory concentration (CMI) of 0.25 µg mL^−1^. Microscopic analysis revealed membrane damage in *Vibrio vulnificus*, including pore formation, loss of cellular content, and the development of cell cavities. Structural analysis identified the active compound as amicoumacin A. Similarly, *B. pumilus* UMA169, isolated from the intestinal tract of sea bream (*Sparus aurata*) fed with a blend of microalgae, demonstrated probiotic potential at a concentration of 145 µg mL^−1^ due to its antagonism toward several fish pathogens [132]. The extracellular products (ECPs) produced by UMA169 showed microalgal degradation capability, and dietary supplementation of these ECPs enhanced digestive enzyme activity in *S. aurata* [105]. The *B. pumillus* UMA216 strain was isolated from the gut microbiota of *Sparus aurata* specimens. The ECPs of this strain have shown in vitro properties, like proteolytic activity and antimicrobial activity against *Photobacterium damselae* subsp. *piscicida*, *Vibrio harveyi*, and *Tenacibaculum maritimum* at a CMI of 114 µg mL^−1^ [105].

*Bacillus subtilis* COFCAU_BSP3, isolated from the intestine of *L. rohita*, showed potent antimicrobial activity along with desirable probiotic traits, such as strong adhesion, surface hydrophobicity, auto-aggregation, extracellular enzyme production, antioxidant activity, and non-hemolytic behavior [133]. Its cell-free supernatants functioned effectively as in vitro postbiotics, exhibiting antibacterial, antibiofilm, anti-virulence, immunomodulatory, and biosafety properties [128]. Additional *B. subtilis* strains have also been isolated from deep-sea sediment and water samples, and their ethyl acetate extracts demonstrated antimicrobial activity against selected pathogens at 5 mg mL^−1^ [134].

*Bacillus velezensis* Z01, isolated from a brackish water sample, produces a variety of bioactive compounds, including antibiotics, antioxidants, antifungal metabolites, and exopolysaccharides (EPS), and possesses the biosynthetic pathway for riboflavin production [135]. Z01 also expresses a serine metalloprotease, Velefibrinase, which lacks hemolytic and hemorrhagic activity and shows high specificity for fibrin substrates. The structure of native Velefibrinase, purified by DEAE-Sephadex chromatography, was analyzed using circular dichroism (CD) spectroscopy. The CD spectrum showed two positive peaks at 192 nm and 196 nm, and two negative peaks at 194 nm and 221 nm. In vitro, Velefibrinase exhibited thrombolytic, antiplatelet, and coagulation-modulating effects, suggesting its potential clinical application as a thrombolytic agent; it has been observed that this compound exhibits these activities starting from the concentration of 0.2 µM [136].

*Halobacillus salinus* C42, isolated from a seagrass sample, produces two phenethylamide derivatives—*N*-(2′-phenylethyl)-isobutyramide and 2,3-methyl-*N*-(2′-phenylethyl)-butyramide—that inhibit *quorum sensing* in *Chromobacterium violaceum* CV026, *Vibrio harveyi* BB120, and *E. coli* JB525 [137]. Both metabolites were purified using a combination of column chromatography with silica gel and thin-layer chromatography (TLC). Structurally, *N*-(2′-phenylethyl)-isobutyramide features an isopropyl group attached to the carbon adjacent to the carbonyl, whereas 2,3-methyl-*N*-(2′-phenylethyl)-butyramide has a sec-butyl group at the same position.

*Streptomyces vinaceusdrappus* AMG31, obtained from marine samples, produces ECPs with potent antioxidant and anti-inflammatory effects through the inhibition of 5-lipoxygenase (5-LOX) and cyclooxygenase-2 (COX-2) [138]. Exopolysaccharides (EPSs) also display anti-Alzheimer’s and anti-obesity activities by inhibiting butyrylcholinesterase and pancreatic lipase, respectively. Furthermore, EPSs inhibited α-amylase and α-glucosidase, mimicking acarbose’s antidiabetic mechanism. The compounds also exhibited broad-spectrum antibacterial and antibiofilm activities against both Gram-positive and Gram-negative bacteria at a concentration of 6.48 µg mL^−1^ [138].

The *Weissella cibaria* strains CECT 30731 and CECT 30732, isolated from the skin mucus and intestine of rainbow trout, have been applied as heat-inactivated postbiotics [139]. Their administration improved the gut microbiota composition by increasing lactic acid bacteria and stimulating IL-1β production, ultimately enhancing survival against *Yersinia ruckeri* infection [139]. Furthermore, *W. cibaria* 17MD and 13ID, isolated from the same host and environment, also exhibited antimicrobial activity against *Aeromonas salmonicida* when applied as supernatant and heat-inactivated cell preparations [140].

In the context of Gram-negative bacteria, certain genera traditionally associated with pathogenicity—such as *Aeromonas* and *Vibrio*—have also demonstrated probiotic and postbiotic potential. *Aeromonas salmonicida* A3-47 and *A. sobria* A3-51, isolated from trout, were shown to antagonize *V. harveyi* in vitro [141]. Outer membrane proteins (OMPs) from these strains, purified according to the method of Lambert, cross-reacted with antibodies against *V. harveyi.* When administered to juvenile trout via oral or intraperitoneal routes, the OMPs stimulated the production of specific antibodies, cross-reacting with *V. harveyi* OMPs, and the non-specific immune responses. The treatment also conferred protection against experimental infection with *V. harveyi* [139]. These findings highlight the potential of OMPs as postbiotics with both antimicrobial and immunogenic properties.

*Halomonas meridian* KKU-MS11, isolated from the Red Sea, produces L-glutaminase, an enzyme with recognized anticancer properties [142]. L-glutaminase plays a crucial role in glutamine-deprivation therapy by catalyzing the hydrolysis of L-glutamine into L-glutamic acid and ammonia. This process selectively inhibits tumor cell growth at concentrations of 7–13 µg mL^−1^ by blocking de novo protein synthesis and inducing oxidative stress through increased superoxide levels, ultimately promoting cancer cell death [143].

*Mameliella* sp. M20D2D8 was isolated from hypersaline waters in South Korea. When an ethyl acetate-soluble extract from the culture was used as a postbiotic, it demonstrated antiviral activity against influenza A and B viruses in MDCK and A549 cells by reducing viral protein synthesis and promoting apoptosis in infected cells. Additionally, the extract showed low cytotoxicity in in vitro assays at 1.42–1.59 µg mL^−1^ [144].

Several strains of *Pseudoalteromonas* have demonstrated promising postbiotic traits. The ECPs of *Pseudoalteromonas flavipulchra* enhance microalgae growth and inhibit *Vibrio* sp. [145]. Interestingly, the activity was lost under heat, alkaline conditions, and protease treatment, suggesting a proteinaceous nature. *Pseudoalteromonas piscicida* S2040, isolated from copepods near the Australian coast, produces a novel siderophore, pseudochelin A, with a 4,5-dihydroimidazole-catechol structure [146]. These mixed-ligand siderophores play a role in bacterial virulence by enabling pathogens to evade host immune defenses while sequestering iron. Pseudochelin A was purified by reversed-phase HPLC, and its chemical structure was elucidated using two-dimensional nuclear magnetic resonance (2D NMR) and tandem mass spectrometry (MS/MS), showing a 4,5-dihydroimidazole moiety and catechol groups. Although pseudochelin A displayed only moderate iron-chelating activity, the antibacterial activity observed in crude extracts is likely due to co-occurring compounds such as myxochelin A and alterochromides, which are recognized for their cytotoxic properties. *Pseudoalteromonas* sp. IBRL PD4.8, isolated from the surface of *Caulerpa racemosa*, produces bioactive compounds that inhibit biofilm formation and growth of *Vibrio alginolyticus* FB3 at 2.78 µg mL^−1^. These compounds were obtained by column chromatography and preparative thin-layer chromatography (TLC) [147]. The microscopic images revealed extensive structural damage to treated biofilms, and the chromatographic mass spectrometry analyses identified a polyunsaturated fatty acid, 4,7,10,13-hexadecatetraenoic acid (C_16_H_24_O_2_), as the main bioactive compound responsible for these effects. This strain shows potential as a natural alternative to conventional antifouling agents. Also, *Pseudoalteromonas haloplanktis* TAC125, isolated from Antarctic coastal waters, is widely used in biotechnology due to its cold-adapted enzymes [148]. A protein derived from its cell-free supernatant used at 10 µM inhibited biofilm formation by *Staphylococcus epidermidis*, a common cause of device-associated infections [149].

The exoproteome of *Ruegeria pomeroyi* DSS-3 is composed by RTX-like proteins, one of which comprises over 50% of the total exoprotein content [47]. The concentration and purification of proteins derived from ECPs was carried out by trichloroacetic acid precipitation. In the *R. pomeroyi* exoproteome, two additional major proteins, YP_165625 and YP_168868, were identified and annotated as secretory type I repeat proteins with C-terminal domains. The 719-amino acid YP_165625 appears to require no post-translational processing, whereas the larger YP_168868, comprising 2164 amino acids, contains seven RTX-like repeats. These secreted proteins play crucial roles in microbial competition, nutrient acquisition, and ecological adaptation.

*Shewanella putrefaciens* PDP11 was isolated from the skin of healthy gilthead sea bream (*Sparus aurata*). This strain has been studied as a probiotic for use in aquaculture, showing the ability to promote growth [150], stimulate the immune system [151], enhance the stress response [152], and protect against experimental infections with the *Photobacterium damselae* subsp. *piscicida* [153]. Moreover, live cells of PDP11, administered at a concentration of 10^9^ CFU g^−1^ of feed for one month, have shown the capacity to modulate the intestinal microbiota [154] and improve skin healing [155]. Given its potential when administered as live cells, its use as a postbiotic has also been recently explored. In vitro, extracellular products of Pdp11s can reduce the titer of nervous necrosis virus (NNV) [156], exert a cytotoxic effect on different fish cell lines [157], and inhibit biofilm formation by bacterial pathogens relevant to fish [48]. Additionally, the effects of including heat-inactivated cells of SpPdp11 in farmed *Solea senegalensis* specimens was evaluated [158], with minor changes observed in the microbiota and immune response.

The *Vibrio proteolyticus* DCF12.2 strain was isolated from the intestinal microbiota of healthy *Dicologlossa cuneata* at the University of Málaga. This strain has demonstrated its probiotic capacity by inhibiting the growth of fish pathogens and activating the specific and non-specific immune response of farmed fish [159]. This strain has also been evaluated as a postbiotic in several studies, showing beneficial effects when it was added after ethanol inactivation to the diet of *Chelon labrosus* at 10^9^ cells kg^−1^ feed, where it improved the growth performance as well as the nutritional characteristics of the muscle and intestine, without causing any pathogenic alteration [160]. Additionally, it was shown to modulate the expression of genes related to stress and the immune system [161]. Moreover, the extracellular products derived from this strain have also been tested as postbiotics in *Sparus aurata*, where they positively modulated the intestinal condition and reduced the expression of inflammation-related genes [162].

**Table 1:** Postbiotics isolated from aquatic bacterial strains, the extraction methods used for their isolation, and their antimicrobial activities.

Strain	Postbiotic	Extraction Method	Biological Activities	Ref.
Gram-positive bacteria				
*Bacillus amyloliquefaciens* COFCAU_P1	ECPs	Centrifugation and filtration	Growth, biofilm, and motility inhibition of *A. hydrophyla*, *Vibrio* spp., and *S. galactiae*	[128]
*B. amyloliquefaciens* MK135790	ECPs	Centrifugation and ethyl acetate extraction	Inhibition of *E. coli*, *B. cereus*, *B. subtilis*, *L. monocytogenes*, *S. aureus*, and *S. enterica typhimurium*	[130]
*Bacillus pumilus* strain H2	ECPs	Cell lysis and sonication	Inhibition of 29 different *Vibrio* strains	[131]
*Bacillus pumilus UMA169*	ECPs	Centrifugation and filtration	Growth inhibition of *P. damselae* subsp. *piscicida*	[105]
*Bacillus pumilus UMA216*	ECPs	Centrifugation and filtration	Growth inhibition of *P. damselae* subsp. *piscicida* and *T. maritimum*	[105]
*Bacillus subtilis* COFCAU_BSP3	ECPs	Centrifugation and filtration	Growth, biofilm, and motility inhibition of *A. hydrophyla*, *Vibrio* spp., and *S. galactiae*	[128]
*Bacillus subtilis*	ECPs	Centrifugation and ethyl acetate extraction	Inhibition of *S. aureus*, *E. coli*, *Klebsiella* sp., *Proteus* sp., and S. *typhi*	[134]
*Bacillus velezensis* Z01	Serine metalloprotease enzyme	Centrifugation, filtration, and circular dichroism analyzer	Thrombolytic activity, antiplatelet effects, and ability to improve blood coagulation	[135,136,163]
*Halobacillus salinus* C42	*N*-(2′-phenylethyl)-isobutyramide 2,3-methyl-*N*-(2′-phenylethyl)-butyramide	Centrifugation and organic solvent extraction	Inhibit quorum sensing (QS) in *C. violaceum* CV026, *Vibrio harveyi* BB120, and *Escherichia coli* JB525	[137]
*Streptomyces vinaceusdrappus* AMG31	EPS	Centrifugation and alcohol precipitation	Anti-inflammatory effects, anti-Alzheimer’s activity, anti-obesity potential, and antidiabetic properties. Antibacterial and antibiofilm activity against a broad spectrum of pathogenic bacteria	[138]
*Weissella cibaria* CECT 30731	Heat-inactivated cells	Heat inactivation	Increased survival against pathogens of *Y. ruckeri* and *A. salmonicida*	[139]
*Weissella cibaria* CECT 30732	Heat-inactivated cells	Heat inactivation	Increased survival against pathogens of *Y. ruckeri*	[139]
*Weissella cibaria* W.c.17MD	Heat-inactivated cells	Heat and centrifugation	Growth inhibition of *A. salmonicida*	[140]
*Weissella cibaria* W.c.13ID	Heat-inactivated cells	Heat and centrifugation	Antimicrobial activity of *A. salmonicida* and *Y. ruckeri*	[140]
Gram-negative bacteria				
*Aeromonas salmonicida* A3-47 and *A. sobria* A3-51	OMPs	Centrifugation and sonication	Cross-reacted with antibodies obtained against *V. harvey*	[141]
*Alcaligenes faecalis* MK135791 as CAB38	ECPs	Centrifugation and ethyl acetate extraction	Inhibition of *B. subtilis*, *E. coli*, and *S. entrica typhimurium*	[130]
*Halomonas meridian* KKU-MS11	L-glutaminase enzyme	Supernatant purification	Inhibits tumor cell growth and promotes cancer cell death	[142,143]
*Mameliella* sp. M20D2D8	ECPs	Organic solvent and centrifugation	Antiviral effect against Influenza A and B	[144]
*Pseudoalteromonas flavipulchra*	ECPs	Centrifugation and alcohol precipitation	Inhibition of *Vibrio* pathogens	[145,146]
*Pseudoalteromonas piscicida* S2040	Siderophore (Pseudochelin A)	Organic solvent extraction	Inhibition of *Pseudomonas aeruginosa*	[146]
*Pseudoalteromonas* sp. IBRL PD4.8	Fatty acids derived from ECPs	Organic solvent extraction	Produces antibacterial compounds and inhibits the biofilm of *V. alginolyticus* FB3	[147]
*Pseudoalteromonas haloplanktis* TAC125	ECPS	Centrifugation and filtration	Inhibited biofilm formation by *S. epidermidis*	[148,149]
*Ruegeria pomeroyi* DSS-3	Proteins derived from ECPs	Centrifugation and filtration	Identification of the RTX-like protein with possible functions in interaction, nutrient uptake, toxicity, or defense	[47]
*Shewanella putrefaciens* Pdp11	ECPs	Centrifugation and filtration	Growth, biofilm, and motility inhibition of *Vibrio harveyi*, *Photobacterium damselae* subsp. *piscicida*, and *Vibrio anguillarum*	[49,157]
*Vibrio proteolyticus* DCF12.2	ECPs	Centrifugation, filtration, and ethanol inactivation	Modulate the expression of genes and improve growth performance and nutritional characteristics	[159,161,162]

ECPs: extracellular products.

## 6. Conclusions

In conclusion, although few marine microorganisms are currently used as postbiotics, the marine environment still offers considerable potential for the isolation of new strains with postbiotic potential. Non-living derivatives of bacteria, called postbiotics, provide a hopeful way to change intestinal microbiota, improve food digestibility, and support health in both humans and animals. Additional to this traditional effect knowing, antitumoral effects have also been reported, suggesting that some postbiotic compounds, including short-chain fatty acids, may be able to slow cancer cell proliferation, thereby showing their potential as treatment with medicinal uses.

Although this field is still in its nascent stages, the great genetic and biochemical diversity of marine bacteria provides the possibility of finding new bioactive compounds that could help human and animal health, and that could be used in medicines based on marine postbiotics. Improvements in DNA sequencing have led to significant progress in the understanding of marine microorganisms. This is particularly relevant given that most microorganisms inhabiting these communities are not culturable in laboratory conditions. The increase in sequencing data of proteomics and metabolomics has enabled a much more comprehensive view of the genetic and metabolic potential of the microbial species present in marine ecosystems. This, in turn, opens up new possibilities for the identification and development of new marine postbiotics with applications in health.

## Data Availability

No new data were created or analyzed in this study.

## Abbreviations

The following abbreviations are used in this manuscript:

AGP	Antimicrobial growth promoter
AMR	Antimicrobial resistance
CLPs	Pumilacidin-like cyclic lipopeptides
CFUs	Colony-forming units
ECPs	Extracellular products
EPSs	Exopolysaccharides
LPSs	Lipopolysaccharides
QS	Quorum sensing
SCFAs	Short-chain fatty acids
WHO	World Health Organization
